# Mitochondrial Chaperones and Proteases in Cardiomyocytes and Heart Failure

**DOI:** 10.3389/fmolb.2021.630332

**Published:** 2021-04-15

**Authors:** Zee Chen, Lei Huang, Alexandria Tso, Shijia Wang, Xi Fang, Kunfu Ouyang, Zhen Han

**Affiliations:** ^1^Department of Cardiovascular Surgery, Peking University Shenzhen Hospital, Shenzhen, China; ^2^State Key Laboratory of Chemical Oncogenomics, School of Chemical Biology and Biotechnology, Peking University Shenzhen Graduate School, Shenzhen, China; ^3^Department of Medicine, School of Medicine, University of California, San Diego, La Jolla, CA, United States

**Keywords:** mitochondrial chaperone, mitochondrial protease, heart failure, cardiomyocyte, mitochondrial protein homeostasis, mitochondrial protein folding, mitochondrial protein degradation

## Abstract

Heart failure is one of the leading causes of morbidity and mortality worldwide. In cardiomyocytes, mitochondria are not only essential organelles providing more than 90% of the ATP necessary for contraction, but they also play critical roles in regulating intracellular Ca^2+^ signaling, lipid metabolism, production of reactive oxygen species (ROS), and apoptosis. Because mitochondrial DNA only encodes 13 proteins, most mitochondrial proteins are nuclear DNA-encoded, synthesized, and transported from the cytoplasm, refolded in the matrix to function alone or as a part of a complex, and degraded if damaged or incorrectly folded. Mitochondria possess a set of endogenous chaperones and proteases to maintain mitochondrial protein homeostasis. Perturbation of mitochondrial protein homeostasis usually precedes disruption of the whole mitochondrial quality control system and is recognized as one of the hallmarks of cardiomyocyte dysfunction and death. In this review, we focus on mitochondrial chaperones and proteases and summarize recent advances in understanding how these proteins are involved in the initiation and progression of heart failure.

## Introduction

Heart failure (HF) has been considered a worldwide public health problem, affecting approximately 1–2% of the adult population (Tanai and Frantz, 2015). In general, HF occurs when the heart is not able to supply enough blood and oxygen to peripheral tissues and fails to support metabolic demands due to systolic or diastolic dysfunction (Ponikowski et al., 2016; Kurmani and Squire, 2017; Rossignol et al., 2019). Numerous factors, including cardiac structural defects, rhythm abnormalities, and high metabolic demands, can induce HF development. Risk factors such as hypertension, diabetes, obesity, and aging may all contribute to the development and worsening of HF (Lin et al., 2016; Ponikowski et al., 2016; Kurmani and Squire, 2017; Rossignol et al., 2019). HF is the terminal stage in the development of various cardiovascular diseases. Although significant improvement has been made in the treatment of acute cardiovascular diseases in the past several decades, the prevalence and mortality rates of HF are still increasing, especially as the population is continually aging (Metra and Teerlink, 2017).

In cardiomyocytes, mitochondria are not only powerhouses providing more than 90% of the ATP for cell contraction, but also involved in many intracellular signaling processes, including regulation of cytosolic Ca^2+^ concentration, lipid metabolism, ROS production, and apoptosis (Pagliarini and Dixon, 2006; Chandel, 2014, 2015). Mitochondrial dysfunction or abnormalities have been recognized as some of the central characteristics of HF, and mitochondria are considered a powerful therapeutic target in various cardiovascular diseases, including HF (Brown et al., 2017; Dietl and Maack, 2017). Mitochondria possess their own DNA that only encode 13 proteins, and most mitochondrial proteins are encoded by nuclear DNA (Mercer et al., 2011). Nuclear-encoded mitochondrial precursor proteins use corresponding transport and sorting mechanisms to arrive at their appropriate destinations within the mitochondria, where they are further processed and formed into functional assemblies (Chacinska et al., 2009; Varabyova et al., 2013).

On the other hand, non-functional or damaged mitochondrial proteins are degraded to maintain the health of the mitochondria (Fang et al., 2017). Maintenance of mitochondrial protein homeostasis is coordinated primarily by chaperones, which are responsible for the folding, maturation, and activation of newly imported proteins and the refolding of misfolded proteins to prevent formation of toxic protein aggregates (Taipale et al., 2010; Hartl et al., 2011; Penna et al., 2018). Proteases also help maintain mitochondrial protein homeostasis by degrading non-assembled proteins and eliminating misfolded or damaged proteins (Cadete et al., 2019). The induction of heat shock proteins (HSPs) is dependent on the activation of a family of transcription factors, the heat-shock factors (HSFs) which bind to the heat-shock element (HSE) in the promoters of the genes encoding HSPs (Akerfelt et al., 2010; Stephanou and Latchman, 2011). In addition, mitochondrial stress responsive factors, including ATF4, ATF5, and CHOP, and other transcriptional factors, including STATs and p53, may also promote the expression of mitochondrial HSPs or proteases (Stephanou and Latchman, 2011; Melber and Haynes, 2018). Perturbation of mitochondrial protein homeostasis precedes dynamic and cellular mitochondrial changes and is recognized as one of the hallmarks of cardiomyocyte dysfunction and death (Fang et al., 2017). Because many excellent reviews addressing mitochondrial quality control in HF have already described mitochondrial dynamic and cellular changes in cardiomyocytes and their underlying molecular mechanisms in depth (Campos et al., 2016; Qiu et al., 2019; Tahrir et al., 2019; Fan H. et al., 2020; Ghosh et al., 2020), our review focuses on mitochondrial chaperones and proteases and their roles in the development and progression of HF.

## Mitochondrial Chaperones in Cardiomyocytes and Heart Failure

**FIGURE 1:**
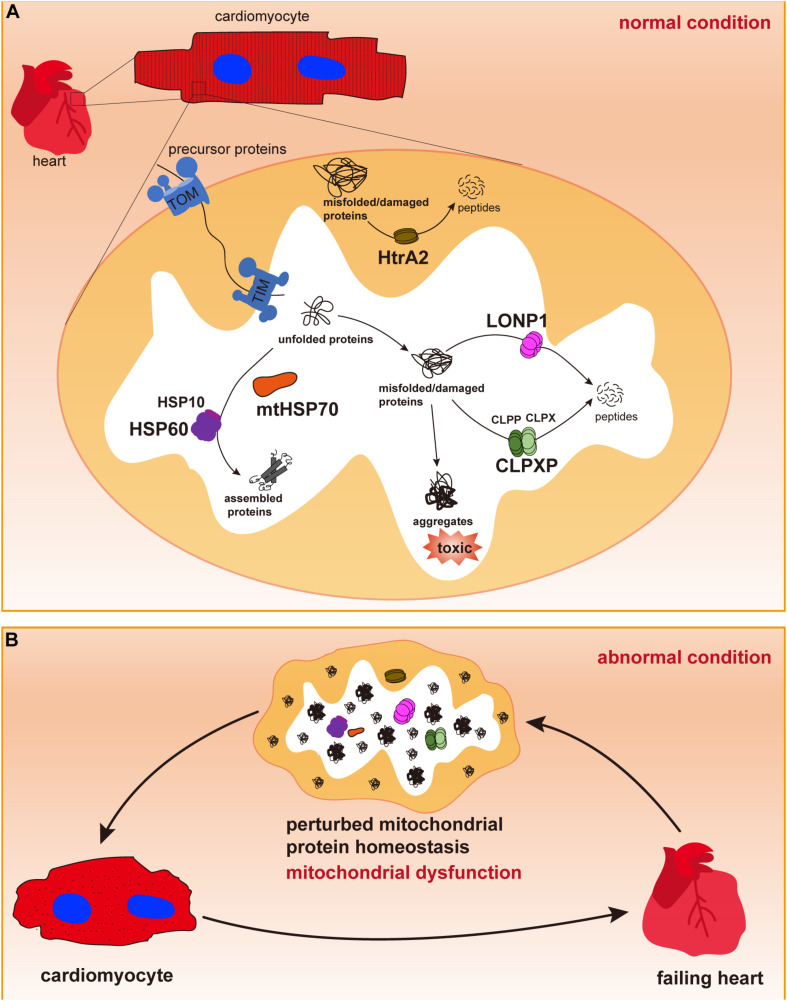
Mitochondrial chaperones and proteases in cardiomyocytes. **(A)** Cardiac cells contain a large number of mitochondria, in which most proteins are synthesized in the cytosol and imported through the outer and inner membranes via TOM and TIM complexes. In the mitochondrial matrix, newly imported and unfolded proteins are folded and assembled to their functional conformations with the assistance of mitochondrial chaperones mtHSP70 and the HSP60/HSP10 complex. Misfolded or damaged proteins are degraded by mitochondrial proteases LONP1, CLPP/CLPX complex in the mitochondrial matrix or by HtrA2 in the intermembrane space to avoid toxic accumulation of protein aggregates. Maintenance of mitochondrial protein homeostasis is essential for normal mitochondrial function. **(B)** Dysregulation of mitochondrial chaperones and proteases may cause accumulation of misfolded/damaged proteins and protein aggregates, thus leading to mitochondrial dysfunction, cardiac cell abnormalities and pathological cardiac remodeling, and heart failure.

Mitochondria possess several groups of chaperones that play crucial roles in maintaining mitochondrial protein homeostasis and function (Voos and Rottgers, 2002). In particular, HSPs, such as HSP60/HSP10 and HSP70, are major chaperone proteins that assist in protein importing, folding, and assembly as well as in the refolding of denatured polypeptides to prevent the formation of aggregates (Figure 1). Next, we summarize the role of these chaperones in regulating cardiac physiology and pathophysiology and discuss how they are involved in HF.

### HSP60 and Its Co-chaperone HSP10

HSP60 is a highly conserved protein in both prokaryotic and eukaryotic cells and is abundantly expressed in cardiomyocytes alongside its co-chaperone HSP10 (Duan et al., 2020). HSP60 is mainly localized in mitochondria, where it is widely recognized as a key player in regulating mitochondrial protein homeostasis (Lin et al., 2007; Duan et al., 2020). HSP60 is also observed in the cytoplasm, plasma membrane, extracellular space, and in the bloodstream, where it may regulate cell apoptosis, proliferation, migration, and immune responses (Lin et al., 2007; Henderson et al., 2013; Meng et al., 2018; Duan et al., 2019, 2020). To facilitate its folding and refolding capacity, the human HSP60 protein forms a symmetrical football complex with HSP10, and its bacteria homolog GroEL forms a barrel-like structure (Ostermann et al., 1989; Horwich et al., 2006; Nisemblat et al., 2015). In cardiac mitochondria, HSP60 plays an essential role in regulating mitochondrial protein homeostasis. Recently, we generated an inducible cardiac-specific HSP60 knockout mouse model and used proteomic analysis to demonstrate that ablation of HSP60 in adult mouse cardiomyocytes caused a downregulation of about 20% in mitochondrial protein levels (Fan F. et al., 2020). These HSP60-dependent mitochondrial proteins are degraded despite being normally imported into mitochondria, which indicates their low stability in HSP60 deficient mitochondria. Furthermore, deletion of HSP60 results in the early onset of mitochondrial unfolded protein response, which is accompanied by changes in mitochondrial complex activities, mitochondrial membrane potential, and ROS production. Together, these mitochondrial abnormalities lead to cell death, cardiac dysfunction, and ultimately caused dilated cardiomyopathy and HF (Fan F. et al., 2020).

On the other hand, overexpression of HSP60 alone or together with its co-chaperone HSP10 in cultured neonatal rat cardiomyocytes has been shown to protect the cells from simulated ischemia and reoxygenation injury (Lau et al., 1997; Lin et al., 2001). Following cardiac ischemia/reperfusion injury, mitochondrial dysfunction and damage are both critical determinants of cell death (Ramachandra et al., 2020; Wang et al., 2020). It is worth noting that HSP60 can translocate to the plasma membrane and cell surface in failing rat and human hearts and that abnormal HSP60 trafficking may be an early indicator for myocyte loss (Lin et al., 2007). Overexpression of HSP60 or HSP10 decreases both the release of mitochondrial cytochrome c and caspase-3 activity while enhancing ATP recovery and mitochondrial complex activities (Lau et al., 1997; Lin et al., 2001), thus indicating that the protective role of HSP60 is directly associated with improved mitochondrial function. In the myocardium of patients with dilated or ischemic cardiomyopathy, the expression of endogenous HSP60 is also significantly increased (Knowlton et al., 1998; Latif et al., 1999). However, in contrast with the protective role of mitochondrial HSP60, extracellular HSP60 may actually promote a proinflammatory response and exacerbate the progression of HF (Lin et al., 2007).

### Mitochondrial HSP70 and Heart Failure

Mitochondrial HSP70 (mtHSP70), also known as Ssc1, GRP75, PBP74, MOT2, or Mortalin, is a highly conserved HSP70 protein family that is predominantly localized in mitochondria and characterized by an N-terminal ATPase domain and a C-terminal peptide-binding domain (Bhattacharyya et al., 1995; Penna et al., 2018). mtHSP70 may play two major roles in regulating mitochondrial protein homeostasis. First, mtHSP70 regulates the transportation of preprotein precursors from the cytosol into the mitochondrial matrix (Wadhwa et al., 2002), a process that is largely dependent on the interaction between mtHSP70 and the mitochondrial inner membrane protein Tim44 (Wachter et al., 1994; Schneider et al., 1996). Second, mtHSP70 may facilitate the folding of newly imported, neo-synthesized, or misfolded mitochondrial proteins to their native conformations with the assistance of its co-chaperones, thus preventing protein degradation and aggregation (Herrmann et al., 1994; Prip-Buus et al., 1996). At the cellular level, mtHSP70 is considered a key player in regulating the mitochondrial stress response along with aging and programmed cell death and is, accordingly, also involved in the modulation of cellular senescence and immortalization (Liu Y. et al., 2005; Jin et al., 2006; Yaguchi et al., 2007). Knockdown of mtHSP70 in human cells is sufficient to cause alterations in mitochondrial morphology, impaired mitochondrial membrane potential, and increased ROS production (Burbulla et al., 2010). Reduced mtHSP70 function is also associated with the activation of the mitochondrial unfolded protein response, increased susceptibility toward intramitochondrial proteolytic stress, more autophagic degradation of fragmented mitochondria, and less mitochondrial mass. Together, these alterations may increase mitochondrial vulnerability for apoptotic cell death (Burbulla et al., 2014). mtHSP70 may also interact with Parkin, Pink1, and DJ-1 (Burbulla et al., 2014) as mutations in mtHSP70 have been linked to neurodegeneration in Parkinson’s disease (De Mena et al., 2009; Burbulla et al., 2010). On the other hand, increased mtHSP70 expression can be observed in myocardial tissues from patients with chronic atrial fibrillation, which was proposed to be an adaptive heat shock response to restore cellular homeostasis (Kirmanoglou et al., 2004). In fact, a cardiac-specific mtHSP70 transgenic mouse model showed that increased mtHSP70 expression could restore cardiac function and nuclear-encoded mitochondrial protein import, contributing to a beneficial impact on proteome signature and enhanced mitochondrial function in diabetic hearts (Shepherd et al., 2018). However, no loss-of-function studies are reported on the role of mtHSP70 in cardiac physiology and HF. Considering that conventional global deletion of mtHSP70 in mice results in embryonic lethality (Krysiak et al., 2015), generating a mouse model with constitutive or inducible gene deletion in a cardiomyocyte-specific manner will be helpful to address this question.

## Mitochondrial Proteases and Heart Failure

Mitochondria possess an independent proteolytic system that efficiently degrades polypeptides to amino acids in different mitochondrial compartments (Deshwal et al., 2020). As of now, more than 45 proteases have been identified in mitochondria, of which 23 are exclusively localized within mitochondria, and the others may shuttle between the cytosol and mitochondria (Quiros et al., 2015; Deshwal et al., 2020). As a part of the mitochondrial protein quality control system, these enzymes take charge of proteolytic processing, decide the dwelling duration of short-lived regulatory proteins, and degrade misfolded and damaged mitochondrial proteins, ultimately preventing the accumulation of toxic aggregates within mitochondria (Hamon et al., 2015; Quiros et al., 2015). Loss of mitochondrial proteases is shown to disrupt mitochondrial functional integrity and is also associated with a variety of human diseases, including HF (Gakh et al., 2002; Vogtle et al., 2018; Ohba et al., 2020). In general, mitochondrial proteases can be divided into ATP-dependent peptidases, ATP-independent peptidases, and oligopeptidases (Cadete et al., 2019). In particular, mitochondrial ATP-dependent peptidases belong to a AAA (ATPase associated with diverse cellular activities) family of proteases, which in mitochondria, includes four members: Lon protease 1 (LONP1) and caseinolytic peptidase P (CLPP) in the matrix and AFG3L2 and YME1L in the inner membrane (Quiros et al., 2015; Cadete et al., 2019; Puchades et al., 2020). The role of AFG3L2 in cardiomyocytes and HF is not well understood. By contrast, it is shown that YME1L and the stress-activated peptidase OMA1 play a key role in balancing the processing of the dynamin-like GTPase OPA1 in the inner membrane (Ehses et al., 2009; Anand et al., 2014), and their roles in regulating mitochondrial dynamics and HF have been well addressed elsewhere (Wai et al., 2015; Marin-Garcia and Akhmedov, 2016; Li et al., 2020; Ramaccini et al., 2020). Here we summarize the role of LONP1, CLPP, and high-temperature-requirement protein A2 (HtrA2) in cardiomyocytes and discuss how these proteases are involved in HF.

## LONP1 in Heart Failure

LONP is the first identified ATP-dependent serine protease, which has two different isoforms, mitochondrial LONP1 and peroxisomal LONP2, encoded by two different genes in mammals (Bota and Davies, 2016). One important role of LONP1 is to maintain mitochondrial protein homeostasis by recognizing and degrading oxidatively modified proteins, which are highly conserved from bacteria to humans (Bota and Davies, 2002, 2016). A list of LONP1 substrates including specific and non-specific substrates has been identified, among which the identified specific substrates are mitochondrial matrix proteins, including adrenodoxin reductase, cytochrome P-450, aconitase, and mitochondrial transcription factor A (TFAM) (Watabe et al., 1993; Bota and Davies, 2002; Matsushima et al., 2010). In particular, TFAM is required for mitochondrial transcription and may be degraded by LONP1 when it is not bound with mtDNA (Matsushima et al., 2010; Lu et al., 2013). Ablation of TFAM decreases mtDNA content and impairs mitochondrial OXPHOS activity, whereas overexpression of TFAM increases mtDNA content and prevents HF in mice (Filograna et al., 2019). Therefore, LONP1 is proposed to play an important role in regulating the stability of the mitochondrial genome and related transcription processes. In addition, it has been shown that LONP1 can also function as a chaperone and a mitochondrial DNA binding protein (Bota and Davies, 2016).

In addition, it is shown that mutant yeast lacking a functional Lon gene have a decreased ability to process mitochondrial matrix proteins (Suzuki et al., 1994). In mammalian cells, downregulation of LONP1 under certain pathological conditions or by genetic approaches can also cause accumulation of damaged proteins within mitochondria (Bota et al., 2002, 2005). In mouse hearts, LONP1 is proposed to have a protective role in ischemia/reperfusion. Haploinsufficiency of LONP1 (*Lonp1*^±^) increases infarct sizes following ischemia/reperfusion injury (Venkatesh et al., 2019). On the other hand, LONP1 is upregulated in mouse hearts during ischemic preconditioning, and overexpression of LONP1 in cardiomyocytes can significantly reduce cardiac infarction and cell apoptosis in mice (Venkatesh et al., 2019). Upregulation of LONP1 may reduce oxidative damage caused by proteins and lipids, preserve the mitochondrial redox state, and reprogram mitochondrial bioenergetics by reducing complex I activity, all of which cumulatively decrease ROS production and cardiac cell death in LONP1-overexpressing hearts (Venkatesh et al., 2019; Venkatesh and Suzuki, 2020). In addition, a recent study posted as a preprint revealed that cardiac-specific LONP1 deficiency causes mitochondrial fragmentation, aberrant metabolic reprogramming of cardiomyocytes, dilated cardiomyopathy, and HF (Lu et al., 2019), suggesting that LONP1 plays an essential role in regulating mitochondrial dynamics and is required for normal cardiac physiology.

Interestingly, LONP1 also undergoes posttranscriptional modifications during HF. In a mouse model generated to study pressure overload–induced HF, increased carbonylation and tyrosine nitration of LONP1 could be found in mitochondria isolated from the failing hearts, which is associated with a reduction of mitochondrial ATP-dependent proteolytic activity (Hoshino et al., 2014). Introduction of a mitochondria-targeted superoxide dismutase mimetic can recover these oxidative modifications in the LONP1 protein and subsequently improve mitochondrial respiration capacity and cardiac function in the failing hearts (Hoshino et al., 2014). Together, these studies strongly suggest that LONP1 plays a critical role in the development and progression of HF and that endogenous LONP1 may be a potential therapeutic target for HF.

## CLPP and Heart Failure

CLPP is another AAA+ protease localized in the mitochondrial matrix, which itself has no ATP-binding ability with very low peptidase activity in the absence of ATP-binding partner CLPX. CLPP is assembled into a double-ringed tetradecameric complex with a hollow chamber containing proteolytic active sites in the presence of its ATPase CLPX (Ishizawa et al., 2019). Targeted proteins are recognized and unfolded by CLPX before being passed on to the CLPP proteolytic chamber for degradation (Sauer and Baker, 2011). CLPP may regulate mitoribosome assembly and, thus, determine the rate of mitochondrial protein synthesis. ERAL1 is a 12S rRNA chaperone that is needed for the formation of functional ribosomal 28S subunits but must be removed prior to the assembly of functional mitoribosomes (Dennerlein et al., 2010; Uchiumi et al., 2010). ERAL1 is recognized as a CLPP substrate (Szczepanowska et al., 2016), and loss of CLPP impairs ERAL1 degradation in cardiac mitochondrial and its dissociation from the small ribosomal complex, thus resulting in accumulation of ERAL1 and reduced amounts of fully formed mitoribosomes, which ultimately disrupts mitochondrial protein synthesis (Szczepanowska et al., 2016).

CLPP deficiency has been linked with severe phenotypes in both mice and humans. Recessive CLPP mutations are observed in the human Perrault syndrome, which is characterized by ovarian failure and sensorineural hearing loss (Jenkinson et al., 2013; Theunissen et al., 2016). CLPP knockout mice exhibit auditory deficits and complete female and male infertility (Gispert et al., 2013). In addition, reduced prenatal/postnatal survival, growth retardation, impairment of movement, and moderate respiratory defects were also observed in CLPP knockout mice (Gispert et al., 2013; Szczepanowska et al., 2016), suggesting that CLPP may play various physiological roles in mammals. However, mice with CLPP deletion specifically in the heart and skeletal muscles survive well without developing any baseline cardiac abnormalities (Seiferling et al., 2016). These muscle-specific CLPP deficient mice do not exhibit any changes in cardiac fetal gene expression, cardiac fibrosis, and UPR^mt^ activation in the heart (Seiferling et al., 2016). Instead, deletion of CLPP may be protective in the heart. In a mouse model with muscle-specific deletion of the mitochondrial aspartyl aminoacyl-tRNA synthetase (DARS2), which is essential for mitochondrial protein translation, deletion of CLPP can significantly attenuate cardiac phenotypes induced by DARS2 ablation (Seiferling et al., 2016). CLPP deficiency may partially restore mitochondrial protein synthesis, increase mitochondrial respiratory activity, reduce pathological cardiac remodeling, and prolong the lifespan of muscle-specific DARS2 knockout mice. Interestingly, deletion of CLPP does not alter the UPR^mt^ induced by DARS2 deletion (Seiferling et al., 2016), implying that CLPP is neither required for nor regulates the UPR^mt^ in mouse hearts. Although the exact molecular mechanisms underlying the protective role of CLPP deletion in DARS2 knockout mice remain unclear, this study suggests that CLPP could be a therapeutic target for treating mitochondrial cardiomyopathy. It would also be worthwhile to test whether CLPP deficiency could play protective roles in other heart disease models, such as pressure overload or ischemia-induced HF.

## HtrA2 in Heart Failure

Although LONP1 and CLPP are localized in mitochondrial matrix, HtrA2, also called Omi, is a serine protease that is localized in the mitochondrial intermembrane space (Goo et al., 2013). HtrA2 also plays a significant role in removing denatured proteins from mitochondria following heat shock or other stress (Martins et al., 2003). Loss of HtrA2 protease activity by a missense mutation in mice causes accumulation of misfolded and damaged proteins in mitochondria and leads to mitochondrial dysfunction (Jones et al., 2003). These mutant mice develop severe muscle wasting, neurodegeneration, involution of the spleen and thymus, and premature lethality (Jones et al., 2003). Furthermore, a lack of coordination, impaired mobility, and tremors can be observed in HtrA2 knockout mice, resembling the clinical characteristics of human Parkinson’s disease (Martins et al., 2004). Overexpression of wild-type HtrA2 proteins in the central nervous system of HtrA2 mutant mice prevents neurological abnormalities and early death but not premature aging phenotypes, such as weight loss, hair loss, spine curvature, enlarged cardiac chambers, and death by 12–17 months of age (Kang et al., 2013). Reduced glucose metabolism, elevated autophagosome activity, and increased mtDNA deletions are observed in cardiac tissues of the mutant mice with neuronal overexpression of HtrA2 (Kang et al., 2013). Therefore, these studies strongly suggest that HtrA2 deficiency may directly cause cardiomyopathy. However, a mouse model with cardiac-specific deletion of HtrA2 may be helpful to provide direct evidence in the future.

HtrA2 has also been recognized as a proapoptotic factor. HtrA2 can translocate into the cytosol or the mitochondrial matrix upon heat shock or hypoxia (Kilbride and Prehn, 2013). Myocardial ischemia/reperfusion can significantly increase cytosolic HtrA2 protein levels, cytochrome c release, and cell apoptosis (Liu H.R. et al., 2005). Application of the HtrA2 inhibitor can significantly reduce both caspase 3 and caspase 9 activities, cell apoptosis, and infarction size in both mouse and rat hearts following ischemia/reperfusion injury (Liu H.R. et al., 2005; Bhuiyan and Fukunaga, 2007), indicating that HtrA2 translocated from mitochondria to the cytosol may promote cardiac cell apoptosis in a caspase-dependent pathway. In fact, overexpression of HtrA2 in mouse cardiomyocytes is sufficient to induce activation of caspases, cell apoptosis, cardiac dysfunction, and pathological cardiac remodeling *in vivo* (Wang et al., 2016). Cardiac cells overexpressed with HtrA2 are also more sensitive to hypoxia and reoxygenation-induced apoptosis (Wang et al., 2016). Together, these studies show that inhibiting HtrA2 may be a therapeutic approach to ameliorate cardiac dysfunction in ischemic HF. Furthermore, HtrA2 may be a potential biomarker for the identification of ischemia/reperfusion injury because the levels of Htra2 in blood serum are significantly increased in mice with myocardial ischemia/reperfusion injury and also in human patients with ST-segment elevation myocardial infarction (Hortmann et al., 2019).

## Conclusion

Mitochondrial chaperones and proteases play an essential role in maintaining mitochondrial protein homeostasis with the former controlling mitochondrial protein folding and refolding and the latter governing mitochondrial protein processing and degradation. Dysregulation of these proteins may perturb mitochondrial protein quality control and cause various defects in both cellular and animal levels. Because mitochondrial chaperones and proteases all have diverse and numerous substrates, the precise molecular mechanisms underlying how these proteins regulate cardiac physiology and pathology still remain largely elusive and require more investigation, such as a combination of multi-omics analysis with *in vivo* animal models. However, it is now clear that mitochondrial chaperones and proteases are required for maintaining normal mitochondrial function and cardiac physiology and that these proteins are also involved in the initiation and progression of HF. Furthermore, the evidence also supports the idea that certain members of mitochondrial chaperones and proteases can either be biomarkers or therapeutic targets for heart diseases such as HF.

## Author Contributions

ZC, LH, AT, SW, XF, KO, and ZH wrote the manuscript. All authors contributed to the article and approved the submitted version.

## Conflict of Interest

The authors declare that the research was conducted in the absence of any commercial or financial relationships that could be construed as a potential conflict of interest.
